# Evaluation of the implementation process of the mobile health platform ‘WelTel’ in six sites in East Africa and Canada using the modified consolidated framework for implementation research (mCFIR)

**DOI:** 10.1186/s12911-021-01644-1

**Published:** 2021-10-26

**Authors:** Samia El Joueidi, Kevin Bardosh, Richard Musoke, Binyam Tilahun, Maryam Abo Moslim, Katie Gourlay, Alissa MacMullin, Victoria J. Cook, Melanie Murray, Gilbert Mbaraga, Sabin Nsanzimana, Richard Lester

**Affiliations:** UBC: The University of British Columbia, Vancouver, BC Canada

**Keywords:** mHealth, Virtual health, Implementation science, Consolidated framework for implementation research, WelTel, Global health

## Abstract

**Background:**

Health systems globally are investing in integrating secure messaging platforms for virtual care in clinical practice. Implementation science is essential for adoption, scale-up, spread and maintenance of complex evidence-based solutions in clinics with evolving priorities. In response, the mobile Health (mHealth) Research Group modified the existing consolidated framework for implementation research (CFIR) to evaluate implementation of virtual health tools in clinical settings. WelTel® is an evidence-based digital health platform widely deployed in various geographical and health contexts. The objective is to identify the facilitators and barriers for implementing WelTel and to assess the application of the mCFIR tool in facilitating focus groups in different geographical and health settings.

**Methods:**

Both qualitative and descriptive quantitative approaches were employed. Six mCFIR sessions were held in three countries with 51 key stakeholders. The mCFIR tool consists of 5 Domains and 25 constructs and was distributed through Qualtrics Experience Management (XM). “Performance” and “Importance” scores were valued on a scale of 0 to 10 (Mean ± SD). Descriptive analysis was conducted using R computing software. NVivo 12 Pro software was used to analyze mCFIR responses and to generate themes from the participants’ input.

**Results:**

We observed a parallel trend in the scores of Importance and Performance. Of the five Domains, Domain 4 (End-user Characteristics) and Domain 3 (Inner Settings) scored highest in Importance (8.9 ± 0.5 and 8.6 ± 0.6, respectively) and Performance (7.6 ± 0.7 and 7.2 ± 1.3, respectively) for all sites. Domain 2 (Outer Setting) scored the lowest in both Importance and Performance for all sites (7.6 ± 0.4 and 5.6 ± 1.8). The thematic analysis produced the following themes: for areas of strengths, the themes brought up were timely diagnosis and response, cost-effectiveness, and user-friendliness. As for areas for improvement, the themes discussed were training, phone accessibility, stakeholder engagement, and literacy.

**Conclusion:**

The mCFIR tool allowed for a comprehensive understanding of the barriers and facilitators to the implementation, reach, and scale-up of digital health tools. Amongst several important findings, we observed the value of bringing the perspectives of both end users (HCPs and patients) to the table across Domains.

*Trial Registration*: NCT02603536 – November 11, 2015: WelTelOAKTREE: Text Messaging to Support Patients With HIV/AIDS in British Columbia (WelTelOAKTREE). NCT01549457 – March 9, 2012: TB mHealth Study—Use of Cell Phones to Improve Compliance in Patients on LTBI Treatment.

**Supplementary Information:**

The online version contains supplementary material available at 10.1186/s12911-021-01644-1.

## Background

Mobile Health (mHealth) is the provision of health services and healthcare support via mobile devices [1]. The substantial increase in the global mobile phone penetration rate, reaching 90% in 2017 [2], as well as the advancement of mobile technologies, led to the emergence of the mHealth field in 2006 [3]. The incorporation of mHealth into healthcare delivery in the past decade is revolutionary with numerous stakeholders invested in mobile technology for health purposes [4]. Although a number of these interventions have shown efficacy and success in global health settings, a select few have reached scalability [5]. Understanding diverse stakeholders is a key factor to drive effective scale-up and spread (SUS) of mHealth interventions [4, 5]. Implementation science is an emerging field of research that focuses on describing each step of the implementation process of a health intervention with emphasis on the barriers and facilitators of the innovation aiming to increase in scale in targeted communities [6, 7].

The consolidated framework for implementation research (CFIR) is a framework developed to assess the effectiveness and efficacy of the implementation process of health interventions across different stages of implementation [6]. In 2015, a mobile health specific version of the CFIR, the modified CFIR (mCFIR), was developed by the Mobile Health Research Group at the University of British Columbia (UBC) to facilitate formative and summative evaluation of mobile health interventions and guide future practices and scale up of interventions, please refer to additional file 1 [8]. The mCFIR reframed the constructs from the perspective of mHealth. A scoring system was added for each construct to rate the Importance and Performance of the various aspects of the implementation process.

WelTel was the world’s first digital health platform to utilize text messaging between patients and providers and first to demonstrate improvement in health outcomes [9]. For the past 15 years, WelTel has been implemented in various health contexts in East Africa, the United States, and in Canada, within both urban and rural communities [10, 11]. In Northern Kenya, WelTel has been used to address suboptimal access to health services including vaccination access and antenatal care [7]. In Rwanda, WelTel has been implemented in Human Immunodeficiency Virus/Acquired Immunodeficiency Syndrome (HIV/AIDS) clinics to improve adherence to antiretroviral therapy and enhance patient engagement with care [7]. In Canada, WelTel has been implemented for HIV, asthma, and tuberculosis care, and in rural areas to support primary care clinics.

This paper examines the implementation of WelTel across six sites, three being in East Africa and three in Canada. The objectives are: (1) to identify the facilitators and barriers to the implementation of WelTel in relation to scale up, and (2) to assess the experiences of using the mCFIR to collect and analyze data of focus group discussions, and to provide a guide for mHealth researchers and implementers on how to use the mCFIR tool for digital health implementations.

## Methods

### Overview of intervention

WelTel is a digital health communication tool that allows patients to communicate with healthcare providers (physicians, nurses, public health officers, etc.) via short message service (SMS), voice and video call. The WelTel tool is based on an open-ended ‘check-in’ model, based on an “Ask, Don’t Tell” approach, in which patients receive a SMS asking them “How are you?” [12]. Patients can respond at any time and reach a health care provider (HCP). In turn, responses are automatically sorted into categories for the HCP to review and triage. HCPs can respond via the dashboard using SMS, phone, or video. HCPs can utilize other patient support and data collection features present within WelTel.

### Overview of intervention sites

A total of six sites were selected for the implementation research study. Two of the sites are in Samburu County in rural Kenya, one in Kigali, Rwanda, two in Vancouver, Canada, and one in Haida Gwaii, a remote island on the Northwest Coast of British Columbia (BC), Canada. The following is the description of each of the sites to understand the context of the implementations. Table 1 captures the timeline of each of the implementation sites.

**Table 1:**
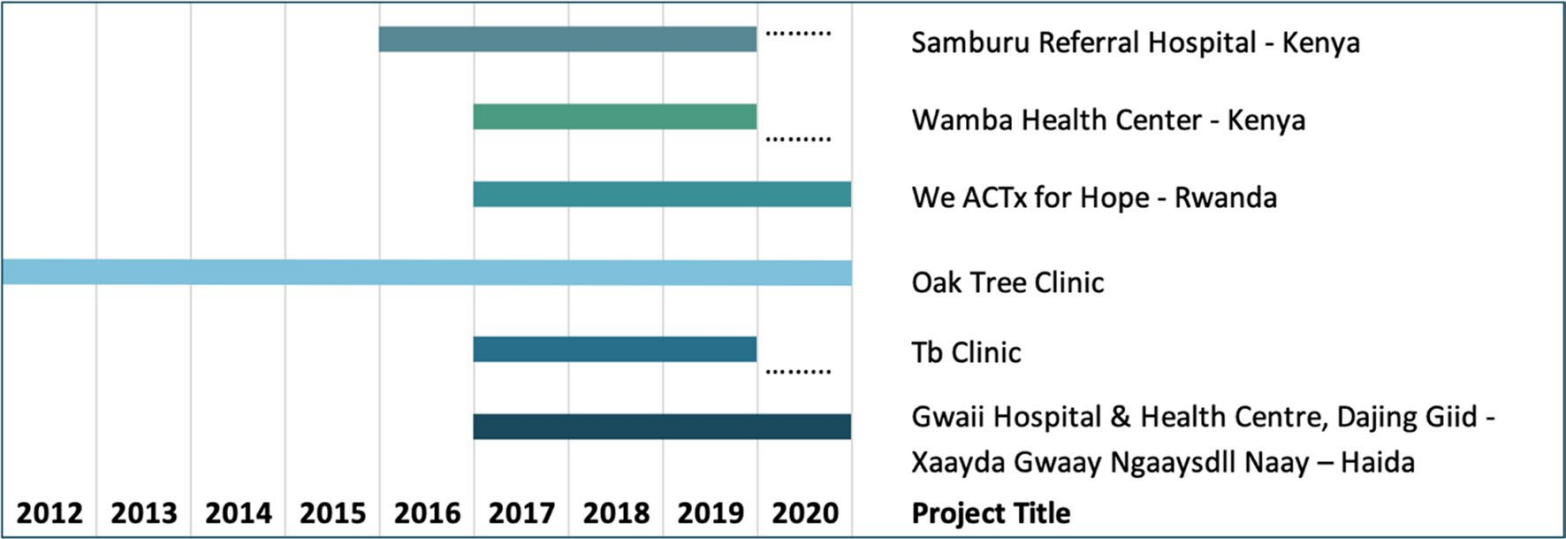
WelTel project gantt chart

#### Maralal Referral Hospital, Kenya

It is one of the largest hospitals in Samburu County, located in Kenya’s vast Northern Arid Lands. The hospital offers a range of services to thousands and caters to the rural population of Samburu Country, with a population of around 310,000 [13], where patients, including many traditional pastoralists, travel long distances for the procurement of healthcare services. According to the 2014 Kenya Demographic and Health Survey, 52.7% of deliveries in Samburu were attended by a friend or relative, whereas only 29.1% deliveries were attended by skilled practitioners [10]. In July 2016, WelTel was introduced to Maralal Referral Hospital and integrated into the antenatal care clinics (ANC), immunization clinics (IMMC), and comprehensive care clinics (CCC). WelTel was implemented with the goal of improving patient engagement and adherence to treatment [7]. Since 2016, around 2000 patients have been enrolled to the platform and followed up for adherence.

#### Wamba Health Center (WHC), Kenya

WHC is located in Wamba, a rural town in Samburu County. WHC provides inpatient and outpatient health services to the population (~ 10, 000) living in and surrounding Wamba [14]. Based on positive outcomes observed in Maralal, in September 2018, WelTel was scaled up to two other clinics in WHC. Since then, around 500 patients have been enrolled on the platform. WelTel has been implemented in maternal, neonatal, and child health programs.

#### We ACTx for Hope, Rwanda

WE-ACTx is a local community-based HIV/AIDS initiative in the capital city of Rwanda, Kigali [11]. The project was launched in 2004 in response to an urgent global appeal from Rwandan genocide survivors to access HIV/AIDS medications. Today, a dedicated team of Rwandan HCPs operate their own Non-Governmental Organization (NGO) – the WE-ACTx for Hope clinic. WE-ACTx provides HIV care and treatment services to more than 2000 patients in Kigali, including women (67%) and adolescents (20%). In 2017, WelTel was successfully introduced into the clinic with the support of the Rwanda Biomedical Centre (RBC). Over 1000 patients, including youth and key populations, have been enrolled since [15].

#### Tuberculosis (TB) Clinic, Vancouver

A scale-up project was launched following a randomized clinical trial of the use of WelTel for Latent TB Infection (LTBI) at the BC Center for Disease Control [16, 17]. A total of 132 patients were enrolled on the platform between 2017 and 2019.

#### Oak Tree HIV Clinic, BC Women’s Hospital (BCWH), Vancouver

Oak Tree Clinic is a provincial tertiary care center located at the BCWH in Vancouver. The clinic provides multidisciplinary care for women, children and families living with HIV. Between 2012 and 2020, a pilot program was conducted at the Oak Tree Clinic to explore the use of WelTel as a digital health tool where a repeated measures study found the intervention improved viral suppression among vulnerable populations [18–20]. A total of 106 patients have been enrolled to the platform.

#### Xaayda Gwaay Ngaaysdll Naay – Haida Gwaii Hospital and Health Centre, Dajing Giids – Queen Charlotte, Haida Gwaii

Haida Gwaii is an island located off the Northwest Coast of BC, the traditional territory of the Haida Nation. In April 2017, a pilot of WelTel was conducted at Xaayda Gwaay Ngaaysdll Naay, Haida Gwaii Hospital and Health Centre in Dajing Giids, Queen Charlotte. This hospital is one of two on the island and serves nearly 3000 patients from 5 communities, including the neighboring Haida community of HlG̲aagilda – Skidegate. There are four practices (A – D) and a total of 7 Family Physicians. At 17-months, 138 patients were enrolled in WelTel, utilizing the service for chronic disease management through symptoms management and assessment, data sharing, prescription refills, and appointment scheduling and reminders. WelTel is still being implemented in Haida Gwaii and the number of patients and practices using the platform continues to increase. This is the first application of a bidirectional texting service in BC’s primary care system.

### mCFIR framework

The original CFIR unified 19 published implementation theories to provide researchers with a range of constructs (n = 39) within five Domains to promote effective implementation: *inner setting*, *outer setting*, *intervention characteristics*, *implementation characteristics* and *characteristics of people involved* [6]. The mCFIR tool, developed by the UBC mHealth Research Group, digitalized key constructs within the Domains, introducing a scoring system for Importance and Performance of each construct for the implementation goal that can be re-evaluated over time. The tool consists of five key Domains and two sub Domains adapted in its constructs to include a scoring element for relative comparisons [8]. The Domains are (1) Intervention Characteristics, (2) Outer Settings, (3) Inner Settings, (4a) End-User Characteristics – Health Care Providers, (4b) End-User Characteristics – Patients, and (5) Implementation Process. The subDomains are (1) Goal Attainment Scale, and (2) Impact Assessment. Each Domain consists of a number of topics formulated as questions and each question is followed by the Performance and Importance scale to rate from 0 to 10. For additional information, please refer to additional file 2.

### Study population

The mCFIR tool is designed to be administered in a single group session with a team of stakeholders. The team should include a facilitator as well as at least one participant from each of the following stakeholder category: a person involved in the outer setting (i.e., government, policies, other organizations), a person who is part of the implementation team (i.e. program manager, clinical director), a health care provider, and, a patient.

Inclusion criteria consisted of the following: a policy maker role was assigned to a participant if they were affiliated with health authorities or held a leadership position with relations to policies and/or finance. An external stakeholder role is assigned to a participant who works within the health or mHealth sector pertinent to the health issues the clinic addresses but is not a member of the HCP/clinic. The Implementation Manager is the individual in the clinic who coordinates or manages the WelTel implementation on site. Healthcare providers (HCP) can be either physicians, nurses, or medical social workers who are using WelTel to communicate and follow-up with patients. Patients are the end users of WelTel, the one receiving the messages and calls to their devices. Inability or unwillingness to provide consent and lack of access to a cell phone were considered exclusion criteria. Participants were selected from a diverse group of stakeholders with the help of site leaders in order to have a comprehensive representation of the community (Table 2).

**Table 2:** Number and type of participants per site

	Wamba, Kenya	Maralal, Kenya	Kigali, Rwanda	Oak Tree Clinic, Vancouver, BC	Tb Clinic Vancouver, BC	Haida Gwaii, BC
Policy Maker	1	1	2	1	1	1
External Stakeholder	1	2	4	1	1	0
Patients	2	3	3	1	0	0
Health Care Provider (HCP)	2	3	4	2	2	5
WelTel Implementation Manager	1	1	1	1	1	1
Unclassified	0	0	2	0	0	2
Total	7	10	16	6	5	9

### mCFIR session protocol

The mCFIR tool was imported into Qualtrics Survey Software with electronic and paper copies created for the convenience of the participants with sessions taking between 2 to 3 h. Qualtrics is a secure, Health Insurance Portability and Accountability Act (HIPPA), and UBC approved survey tool used for data collection throughout the university.

First, participants are asked to collectively identify the health issue being addressed through the implementation of WelTel, the mHealth platform used, and the implementation goals the site would like to achieve to reach their desired outcomes. The facilitator presents one construct at a time as a question and invites discussion amongst the stakeholder attendees. A note taker is present to take detailed observational notes and the mCFIR session is audio-recorded if consent is unanimous. After each construct, participants are asked to include their anonymous comments via Qualtrics. Participants are then asked to rate, on scale from 0 to 10, the Performance and the Importance of the construct discussed. This process is repeated for every construct for all Domains of the mCFIR tool. Scores of the Performance and the Importance of each construct are captured through the Qualtrics Survey tool.

A total of four trained facilitators were present to moderate the focus group discussions. Semi-structured informal interviews were held over videoconference with the facilitators to capture their experiences using mCFIR as a tool to facilitate discussions around implementation assessment. The process followed by facilitators and the research team to collect, analyze, and share data with the implementing clinics is described below.

### Pre-mCFIR session

A set of slides were prepared to provide a background on the digital health platform being discussed, the field of implementation science, and the mCFIR tool. A note taker was assigned to assist the facilitators by taking observational notes of the session and discussion. Multiple rounds of mock mCFIR sessions were held with the UBC mHealth Research Group to pilot the tool, the Domain questions, and estimate session length. The team concluded that the mCFIR tool would require approximately 2 h to be completed with 8 stakeholders. The mCFIR surveys were built into Qualtrics survey software. Sites were selected if they were currently implementing the digital health tool of interest. Due to the nature of the mCFIR session, convenience sampling was applied. Patient and HCP participants recruitment was conducted through the clinic staff with the guidance of the medical director of the clinics. External stakeholders and policy makers were either identified by the clinic staff or by the research team staff. In the 3 Canadian sites, only patients participants were given honorariums for their attendance. All participant types in East Africa were given honorarium to compensate for expenses incurred or time spent to attend the session. The sessions were audio recorded if all participants provided consent. Tablets were made available by the research team for the sessions conducted in Canada. The facilitators shared the consent form and survey links with the participants prior to the session for convenience.

### mCFIR session

Written consent was obtained from all participants either prior to the mCFIR session or before the beginning of the session. At the beginning of the session, the facilitator collects the consent forms from the participants, including consent to record the discussion. Afterwards, the participants are asked to introduce themselves, their profession, and experience with the digital health platform being discussed or any other digital health platforms. The facilitator goes through the set of slides to provide background on the purpose of the session. Afterwards, the participants are asked to collectively identify implementation goals the team would like to work on in the upcoming 4 to 6 months. After identifying the goals, the facilitator guides the discussion using the mCFIR tool. One construct at a time is presented in the form of a question. During the group discussion, participants are encouraged to put the survey aside, and share their thoughts with the group. After discussing a certain construct, the facilitators ask the participants to score the Importance and Performance of the construct being discussed anonymously on the Qualtrics survey. This process was repeated for each construct, by order of Domain. At the end of the session, the participants are asked to rate the Goal Attainment and Impact Assessments of their goals and outcomes. The sessions’ duration varied from 2 to 3.5 h. The note taker’s role during the session was to support the facilitator, keep track of time, and take notes of the discussion being held as well as any other relevant observations.

### Post mCFIR session

After the session, the facilitator and note taker meet to reflect on the session, share notes, and develop a summary report of the discussion. The report is intended to be shared with the research team, clinic directors, and participants. Additionally, the report includes a snapshot of the session and major points brought up by the participants. Implementation goals identified by participants are highlighted in the report with the aim to guide implementation activities until the next mCFIR session. The mCFIR session is encouraged to be held every six months to 1 year to reassess the goals identified and identify new goals.

### Data analysis

Both qualitative (thematic analysis) and descriptive quantitative data analyses were conducted. Performance and Importance scores which made up the quantitative data of the mCFIR tool were exported from Qualtrics in comma-separated values, imported into Microsoft Excel (2019), and cleaned and analyzed using R statistical software. Data cleaning was done using qualtRics, tidyverse, and dplyr packages in R. Given the objectives of the study, means were calculated as measures of central tendance together with standard deviations (SD) as measures of dispersion to summarize the Performance and Importance scores of each construct and Domain across sites and participant types. In instances where there were missing scores in the constructs, they were presented in the table as a blank box. Data visualizations using heat maps for both Performance and Importance scores for each construct and Domain across sites and participant type were generated with ggplot2 and ggpubr packages in R.

For qualitative analysis, survey comments were exported from Qualtrics and then imported into NVivo 12 Pro software where thematic analysis was conducted following an inductive or exploratory approach. mCFIR session notes were also imported into NVivo. Audio recordings were transcribed manually and then analyzed in NVivo. The responses were coded, grouped into themes, and divided into two major categories: (1) Strengths and Benefits, (2) Barriers and Suggestions. The mCFIR tool, protocol, and analysis frameworks are made available for site leaders and researchers upon request.

## Results

Six mCFIR sessions were held between August 2019 and January 2020 with four facilitators moderating them. There were 51 participants attending the mCFIR session, where 49 responses were recorded through Qualtrics, and two surveys were either missing or not recorded. mCFIR surveys originally captured on paper were manually inputted into Qualtrics post-session.

More participants attended the mCFIR session in East Africa than in Canada. The majority of participants fell under the end user categories, HCPs and patients. Attendance of the mCFIR session in Rwanda was higher amongst external stakeholders due to high interest in understanding patient-provider experiences for the purpose of scale up. The variability in the type of participants attending each mCFIR session site is reflected in the results. The TB Clinic and Haida Gwaii Hospital had no patient attendance. The patient constructs in the Haida Gwaii session were answered by the attending participants from the perspective of the patient. The Haida Gwaii hospital site cited next steps as holding another separate mCFIR session for the patient participants.

Each site was asked to identify health issues and implementation goals at the beginning of the mCFIR sessions. Most of the goals identified revolved around access to HCPs outside of regular visits and treatment follow-up, please refer to additional file 3.

### Importance and Performance scoring

**Fig. 1:**
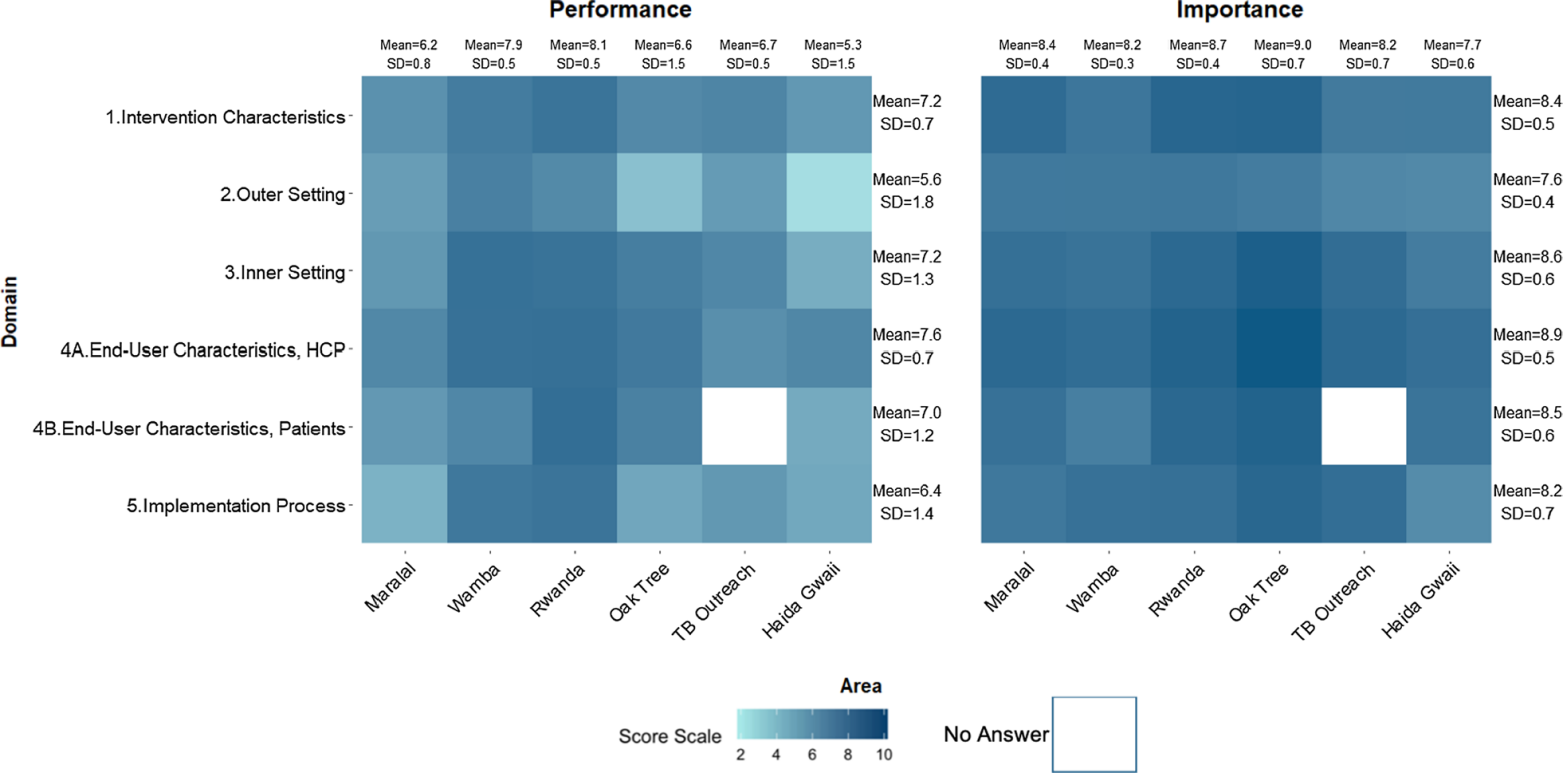
Heat map of the reported Performance and Importance scores per domain and per site

**Fig. 2:**
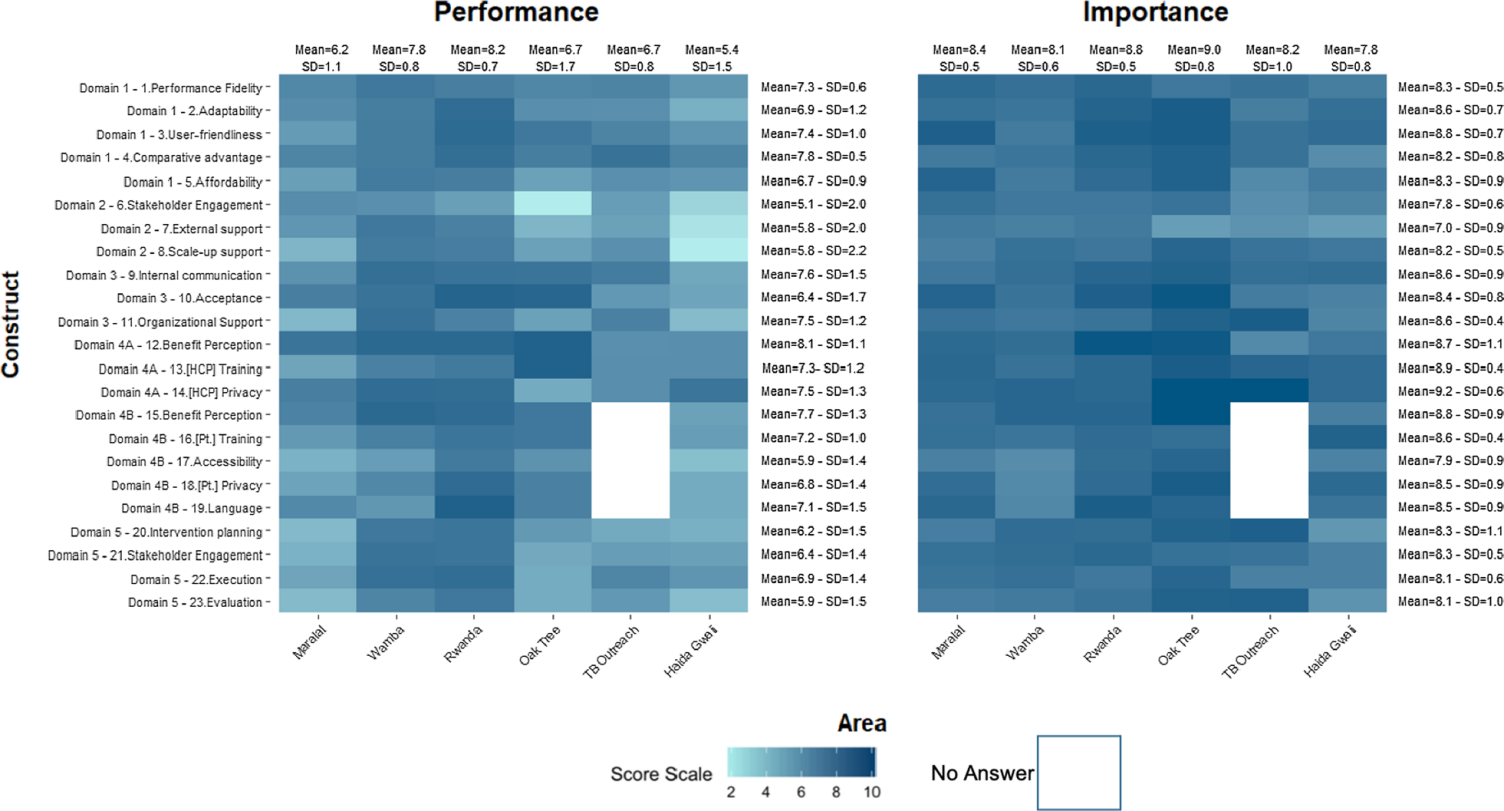
Heat map of the reported Performance and Importance per construct and per site

**Fig. 3:**
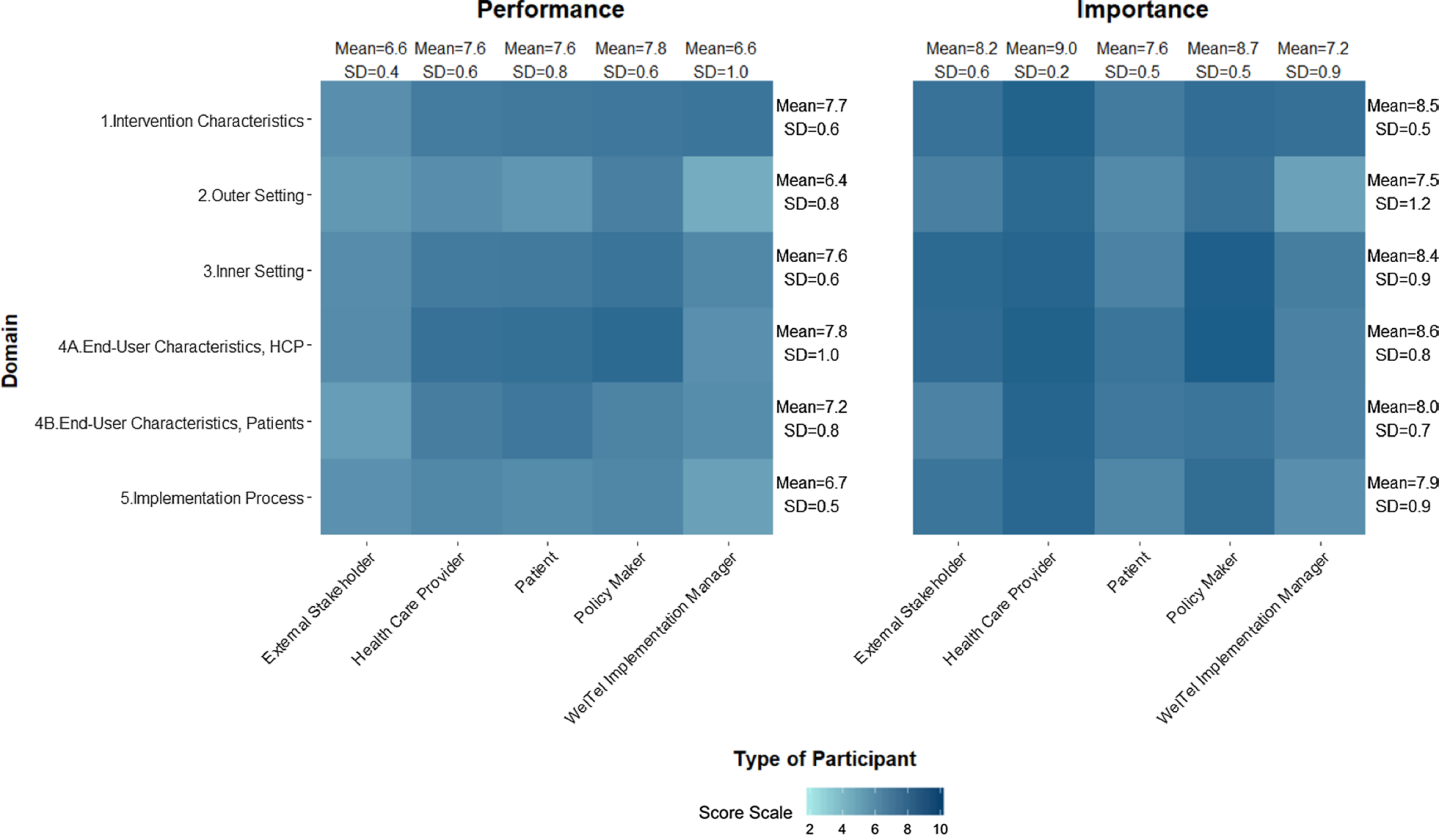
Heat map of the reported scores of Performance and Importance  per domain and per participant type for the East African Sites

**Fig. 4:**
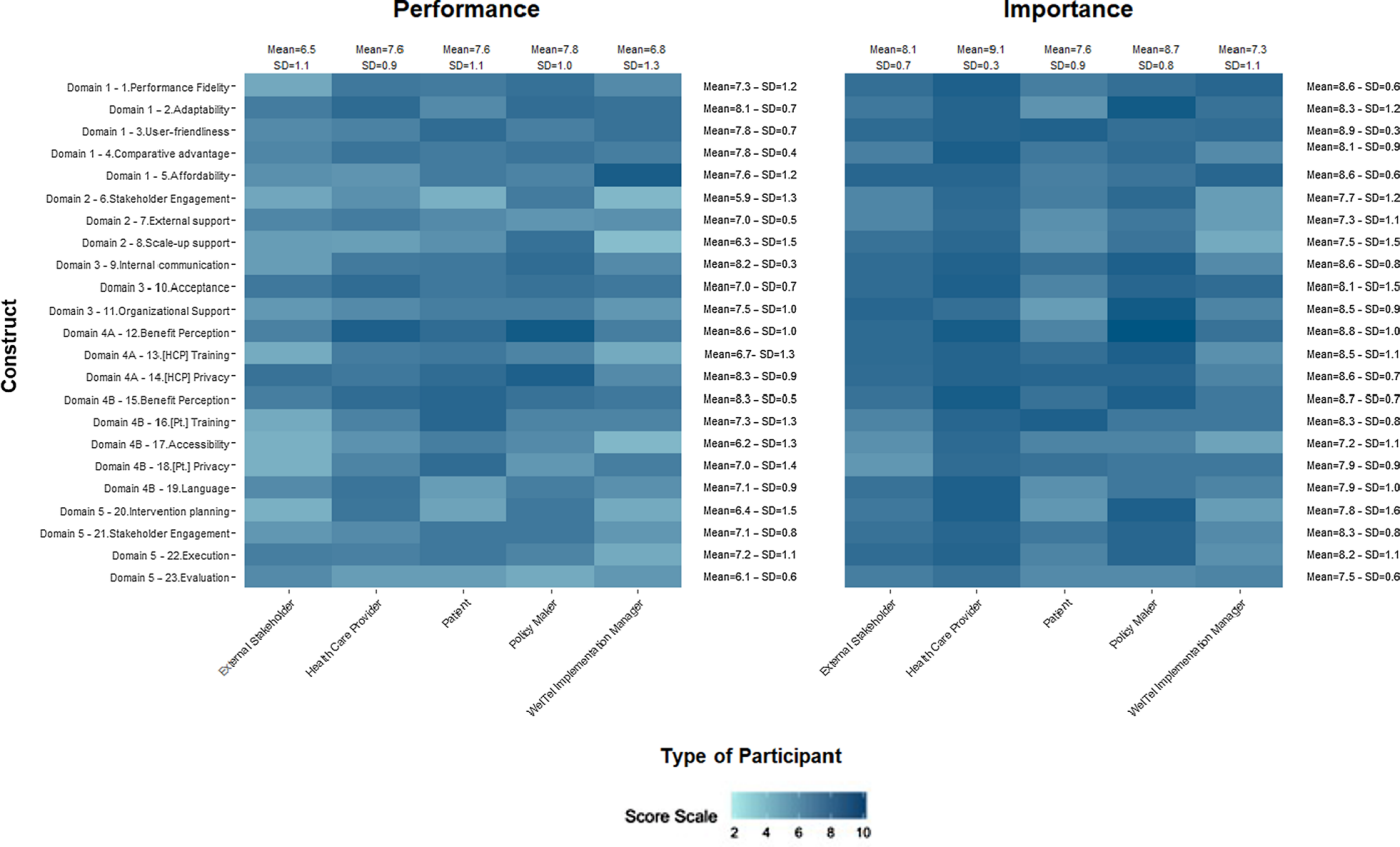
Heat map of the reported scores of Performance and Importance per construct and per site for all three East African sites

**Fig. 5:**
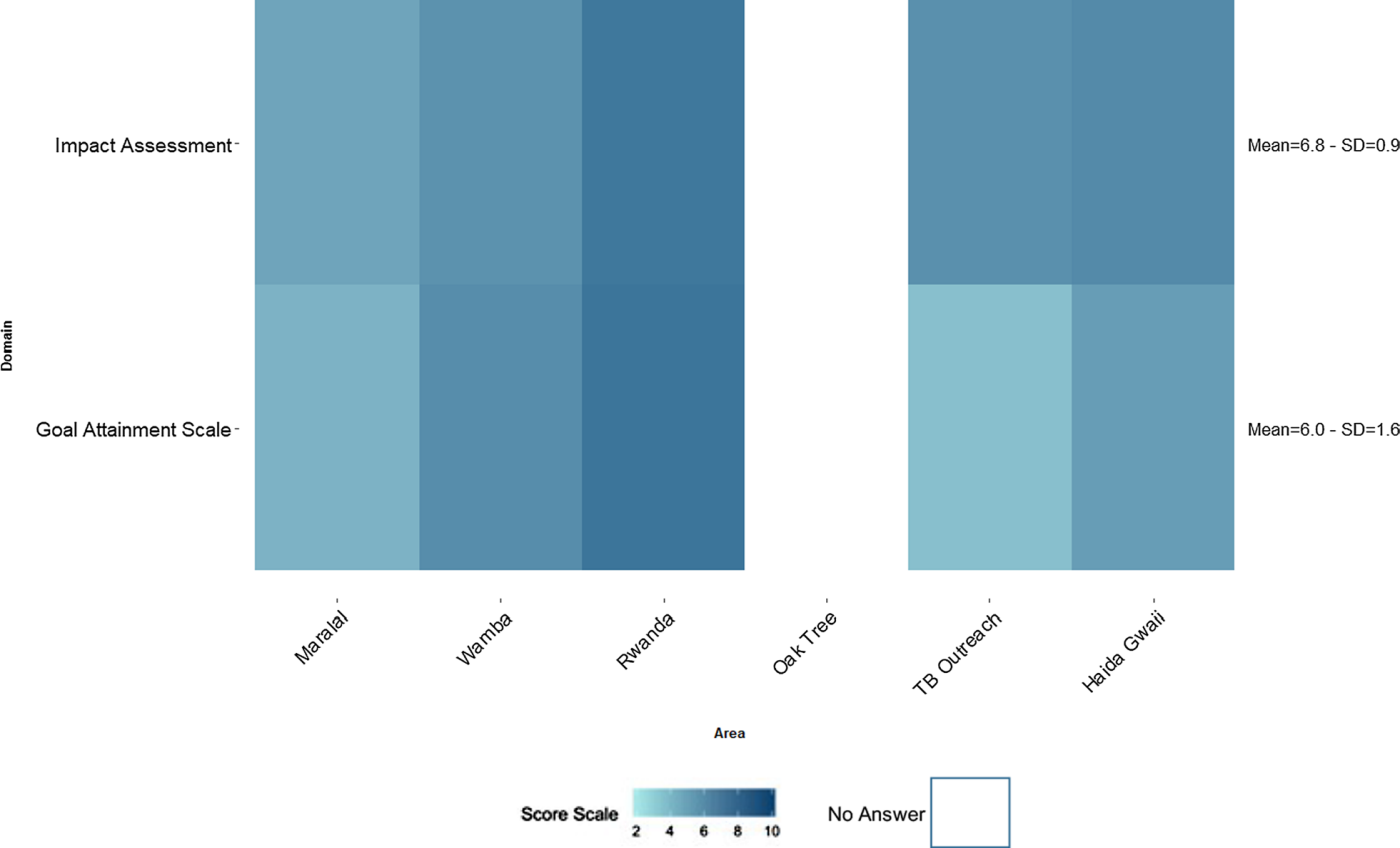
Heat map of the reported scores of Performance for “Goal Attainment” and “Impact Assessment” per site

During the mCFIR sessions, each participant was asked to rate the Performance and Importance of each construct on a scale of 0 to 10 (Figs. 1, 2, 3, 4, 5). The heat map in Fig. 1 presents the scores of Performance and Importance reported per site for each of the five Domains. Scores are displayed following a turquoise spectrum (from pale to dark turquoise). The pale turquoise represents the Domains that scored lowest in terms of Importance and/or Performance, and the dark turquoise represents the Domains that scored highest for Importance and/or Performance.

Overall, participants in the focus groups provided higher scores for Importance as compared to Performance. The mean scores by Domain ranged between 5.6 (SD = 1.8) and 7.6 (SD = 0.7) for Performance and 7.6 (SD = 0.4) and 8.9 (SD = 0.5) for Importnce. Mean scores for both Performance and Importance were highest for the End-user Domain with 7.6 (SD = 0.7) and 8.9 (SD = 0.5) respectively. Similarly, the outer setting Domain had the lowest mean score for both Performance (5.6, SD = 1.8) and Importance (7.6, SD = 0.4).

The highest mean Domain scores for Performance and Importance were from We ACTx and Oak Tree sites respectively. The Haida Gwaii site had the lowest mean Domain scores for both Performance 5.3, SD = 1.5, and Importance 7.7, SD = 0.6 (Fig. 1).

### Performance and Importance scores per construct

Figure 2 represents the rating of the various constructs of each site to better understand which areas act as either facilitators or barriers when implementing WelTel’s platform in each of the intended settings.

The mean Performance scores and Importance in all sites ranged between 5.1, SD = 2.0 and 8.1, SD = 1.1 and 7.0, SD = 0.9 and 9.2, SD = 0.6 respectively. For both Performance and Importance, the highest scoring constructs which were the “benefits perceptions” and “[HCP] privacy” were from the end-user Domain (Domain 4A). Similarly, the constructs with the lowest mean scores for Performance were “stakeholder engagement” and “external support”, both from the Outer Setting Domain (Fig. 2). Notable variations in the mean construct scores were observed across sites. At the TB outreach and Haida Gwaii sites, the “benefit perception” construct did not have the highest mean Performance score as observed in other sites. A similar pattern was observed for Importance where the highest mean construct scores at Maralal, Rwanda, and Haida Gwaii were different from the “privacy” construct that had the highest overall mean score.

### Performance and Importance scores by participant type

Figure 3 presents the reported scores of Importance and Performance per participant type for all three East African sites. The highest Importance ratings were provided by healthcare providers (HCPs) and policy makers, followed by external stakeholders.

By participant type, the mean score for Performance by Domain ranged from 6.6, SD = 1.0 and 7.8, SD = 0.6, and that of Importance ranged from 7.2, SD = 0.9 and 9.0, SD = 0.2. Policymakers rated Performance highest as compared to other participant types with an overall mean of 7.8, SD = 0.6. Policymakers as compared to other participants had similar or higher mean scores for all Domains except Domain 4B – End-UserCharacteristics for Patients. Implementation managers scored Performance of the Outer Setting Domain the lowest. HCPs scored Importance the highest for all Domains in comparison to other participants. Implementation Managers had the lowest scores for all Domains except Intervention Characteristics (Fig. 3).

### High and low constructs for the three East African sites

#### Performance and Importance scores per construct and per participant

The heat map presents the constructs that are perceived as either facilitators or barriers from the perspective of all participant types.

Policy makers provided the highest mean scores for Performance 7.8, SD = 1.0, where “benefit perception” and “healthcare provider privacy” constructs were scored highest in comparison to other constructs. Implementation Managers and External Sakeholders provided the lowest mean scores for Performance, specifically the constructs in the Outer Setting and End User Characteristic Domains.

For Importance, HCPs had the highest overall mean score of 9.1, SD = 0.3, of which they scored highest 14 constructs compared to other participant types (Fig. 5). The constructs scored highest by HCPs were “comparative advantage”, “acceptance”, “benefit perception”, “language”, and “intervention planning”. Alternatively, Implementation Managers had the lowest overall mean score of 7.3, SD = 1.1 where “scale-up support” and “patient accessibility” constructs scored the lowest in terms of Importance.

### Overall performance rated against implementation goals

Figure 5 presents the reported Performance scores for “Goal Attainment” and “Impact Assessment” per site. At the end of the mCFIR sessions, the team revisits the implementation goals identified at the beginning of the session and are asked to rate their overall Performance in achieving their desired goals and outcomes. A total of 5 of the 6 sites filled out these two constructs due to time constraints; with 28 entries recorded for the Goal Attainment scale and 26 recorded for the Impact Assessment. Only half of the participants were able to complete the survey and fill out the last two constructs.

Amongst sites in which the Goal Attainment and Impact Assessment were scored, the overall mean scores were 6.7; SD = 0.9 and 6.0; SD = 1.6 respectively. Of the five sites, Impact Assessment had higher scores when compared to Goal Attainment in three sites: Maralal, TB outreach, and Haida Gwaii. The site in Rwanda had the highest scores for both Domains as compared to the other sites (Fig. 5).

### Qualitative analysis

**Fig. 6:**
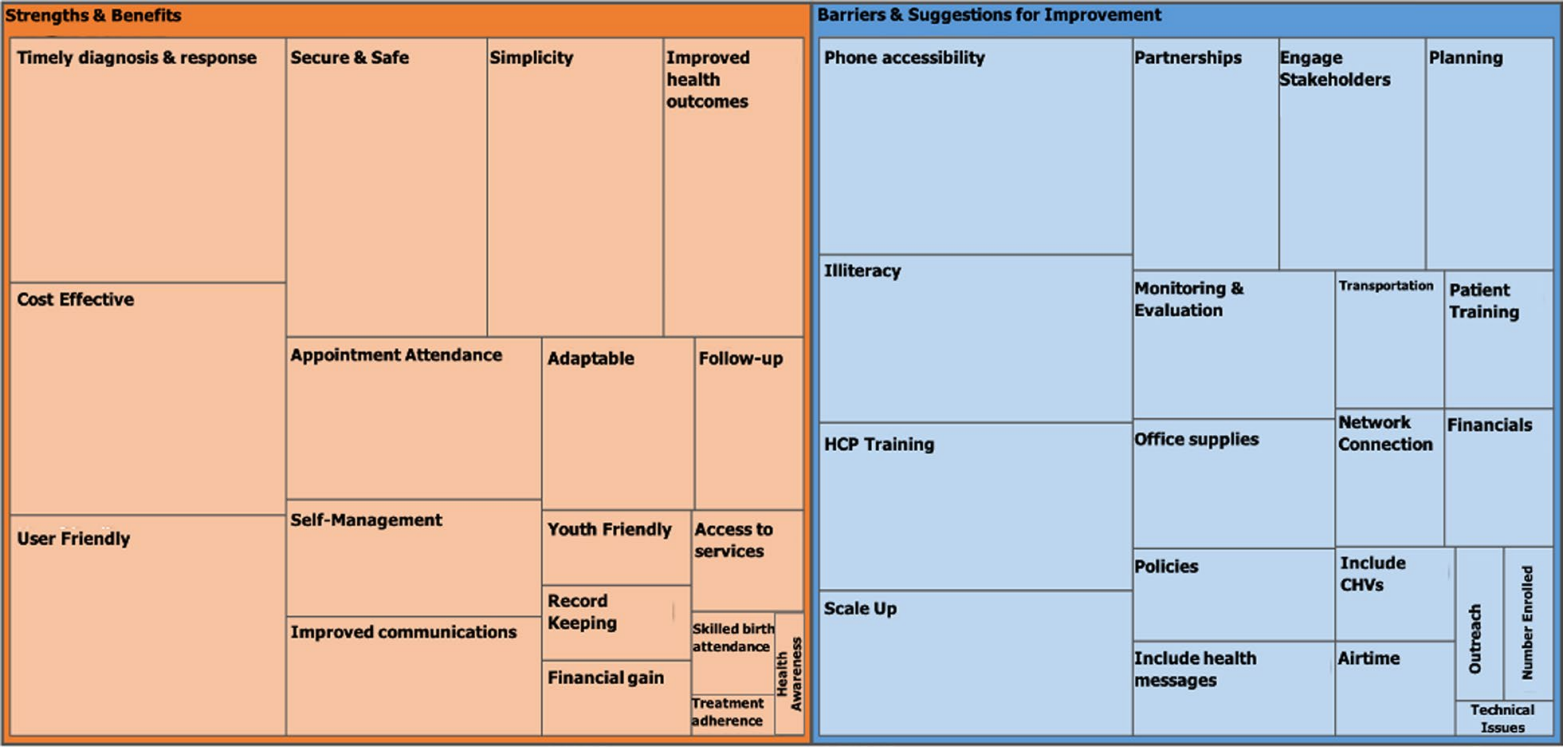
Tree map of the identified Strengths and Barriers to the implementation of WelTel in the 3 East African sites

Participants' inputs during the mCFIR session, summarized in the tree map in Fig. 6, were first divided into two major categories, (1) Strengths and Benefits, (2) Barriers and Suggestions. Subsequently, sub-themes were constructed for each category. The Participants' responses included a combination of evaluation of the intervention (WelTel) and evaluation of the implementation process itself. The larger the area size on the tree map, the greater the proportion. Tables 3 and 4 highlight some of the statements made by the participants during the mCFIR session.

**Table 3:** Reported strengths and benefits to the implementation of the digital health platform WelTel in East Africa

*Timely diagnosis and response*
“It's real time. Being able to communicate if me as a mother to be I do have a problem.” [Patient – Maralal, Kenya]
“Help clients who respond with "Not Okay" while they're at home.” [HCP – Rwanda]
“It has helped many who are in need through SMS [..] which is faster” [HCP – Maralal, Kenya]
“Timely identification of opportunistic infections” [Policy Maker – Rwanda]
“It addresses early referrals and interventions to be taken in case of a patient/client having a complication. The client will text, and the ambulance will go for the patient.” [Policy Maker – Wamba]
“Reference 1: 0.32% coverage
WelTel is directly reaching the beneficiary compared to other existing solutions which is reaching to the beneficiary through CHVs” [External Stakeholder – Wamba]
*Cost-effectiveness*
**“**Less human resources required in comparison to other alternatives” [Policy Maker – Maralal]
“ Is cheap since I don't incur any charges as a client” [Patient – Maralal]
“There is no cost for the users. The cost for the implementer is limited and there is an efficiency in using WelTel.” [Policy Maker – Rwanda]
*User-friendliness*
**“**User friendly to the staff because it’s easy to use and also for the client” [HCP – Wamba]
“Yes—It's a simple tool that even the illiterate can understand.” [HCP– Wamba]
“It is easy for writing the message than talking with the nurse”
“Yes The message only reaches the WelTel personnel from the code 40,540, hence it is private” [Implementation Team Manager – Wamba]
*Security and safety*
**“**Yes…because my privacy is safe” [Client – Maralal]
“The message is coded. Therefore the confidentiality is respected.” [Policy Maker – Rwanda]
“She is the only one who receives the messages from the client/patients” (referring to the Implementation manager) [Client – Wamba]
*Appointment attendance*
“The intervention works well since it reminds clients of when they are supposed to come to the clinic. This has significantly reduced dropouts and improved Skilled Birth Attendance at the facility.” [External Stakeholder – Maralal]
“Yes, this is because through the SMS the client is reminded on when to come for clinics.”
[HCP – Maralal]
“For youth is very acceptable as they don't want to hold program card every time.” [HCP – Rwanda]

**Table 4:** Barriers and Suggestions to the implementation of the digital health platform WelTel in East Africa

*Phone accessibility*
“Some of our homes don't have electricity” [Client – Maralal]
“Sometimes the system is affected by weak network in some selected areas. Some client might not have mobile phones.” [Policy Maker – Maralal]
“it is challenging if you are sharing your phone with somebody.” [Patient – Wamba]
*Literacy*
**“**The illiterate are not confident because they don't know how to respond” [HCP – Maralal]
**“**it become challenge to those that can read and understand.” [Client – Wamba]
**“**Illiteracy level is the only challenge in the community around Wamba therefore confidence among some patients is low” [External Stakeholder – Wamba]
*HCP training*
“Needs more community health volunteers to be trained” [HCP – Maralal]
“We need to train other health workers and CHVs so that everybody can get information about
WelTel. More networks with the community” [HCP – Wamba]
*Scale-up*
**“**Include other health departments like nutrition, Tuberculosis clinic, PNS” [HCP – Maralal]
“WelTel can be adapted in other areas of public health interventions. This includes outbreak
monitoring and control.” [Policy Maker – Maralal]
“Scale it up in areas that really need the services that is with high defaulter rates Think of ways to reduce operation costs in order to allow scale up. The intervention should complement other systems and work towards improving the health systems.” [External Stakeholder – Maralal]
“Communicating WelTel at a national level” [HCP – Rwanda]

### Strengths and benefits

The main sub-themes discussed by participants regarding the first major category, Strengths and Benefits of WelTel's implementation were the following:

1. *Timely diagnosis and response*–Participants discussed the convenience of communicating and addressing health issues in a timely matter from home. A mother from Maralal, Kenya said that the platform “is real-time”, and that she was able to communicate with her HCP whenever she faced a health issue. Policymakers mentioned how the WelTel platform assisted them with “timely identification of opportunistic infections”.
2. *Cost-effectiveness*–Policymakers highlighted the advantage of not requiring additional human resources for the implementation of the digital health platform WelTel. Communicating with patients through the platform has been incorporated into their care process. Patients did not incur any costs when texting their HCPs which has been considered by the patients, a motivation for enrollment.
3. *User-friendliness*–The implementation team managers and other end-users including clinicians reported the ease of using the platform. Patients did not require training as they only needed to reply via SMS to the incoming SMS check-in texts.
4. *Security and safety*–Patients are the only ones who understand that the intentionally ambiguous “How are You?” message is from their HCP. Patients highlighted how their privacy is respected since the language of the message does not disclose their health status.
5. *Appointment attendance*–The use of WelTel texting service to remind patients of their appointments has been highlighted as a benefit by both external stakeholders and patients as it reduces consequences related to loss of patients’ appointment health cards and thus increases attendance.

### Barriers and suggestions

As for the second major category, Barriers and Suggestions, several sub-themes emerged. Issues regarding phone accessibility, literacy, partnerships with stakeholders, staff training, and scale-up of the program were discussed as major barriers to the implementation of WelTel:

1. *Phone accessibility*–Some patients share phones with a family member. This has been highlighted as a potential barrier as these patients might not be reached at all times.
2. *Literacy*–Literacy is a challenge amongst certain patient groups. It creates a barrier by hindering patients' ability to text back to the platform and share their issues and concerns.
3. *HCP training*–Further training has been requested by HCPs to independently train new staff members on the digital health intervention being implemented.
4. *Scale-up*–Participants from the East African sites expressed the desire to scale up the project to other health departments and regions.

## Discussion

This paper focused on identifying the facilitators and barriers to the implementation of WelTel in six sites in Kenya, Rwanda, and Canada, and in assessing the application of a modified consolidated framework for implementation research (mCFIR) tool in facilitating focus groups for mHealth, digital clinical messaging, and virtual care. The CFIR framework was modified to meet the needs of the digital health field to inform stakeholders on ways to enhance and scale up the implementation of the digital health tool under investigation. We tested the modified framework (mCFIR) with the WelTel platform projects across diverse geographic and health settings. For the purpose of this paper, a descriptive quantitative and qualitative analyses were conducted.

### Main findings

We observed that constructs that ranked highest on the gradient of Performance are perceived as facilitators to the implementation of WelTel while those with the highest ranking in Importance are perceived as areas for improvement.

By this categorization, the HCP Domain represented a facilitator for all sites, whereas the Outer Setting Domain represented a challenge reported by most sites. Outer setting corresponds to the environment of stakeholder in which the implementation is occurring; challenges within this Domain reflect the ability to scale-up the intervention. Furthermore, the major priority areas of action reported by all sites involved expansion of WelTel across various sites and services, as well as improving health indicators identified by each site. In fact, the utility of these findings is supported in the real-world implementation progress across these sites; the WelTel tool expanded in Rwanda (where overall scores were highest) and is currently implemented as part of the national COVID-19 (coronavirus disease) pandemic response, paused then restarted in Kenya, during the COVID-19 pandemic (where scores were in the middle), and stopped in the TB program in Vancouver (where overall scores were lowest). The strengths and benefits themes discovered through the qualitative analysis of the open group discussion supported the calculated scorings of the mCFIR constructs. These findings are similar to those of a systematic review that explored the adoption of m-Health by healthcare professionals [21]. Another systematic review of sustainability of tele-homecare programs found that perceptions of effectiveness were a facilitator of sustainability similar to benefit perceptions we found in our study [22].

Among the strengths of the mCFIR approach is in its ability to identify, quantify, and visualize areas of strengths and opportunities for improvement. The 0 to 10 scoring component was added so that stakeholder participants can rank the Performance and Importance of each of the Domains and constructs. Moreover, bringing together a diverse group of stakeholders provides an opportunity to discuss different perspectives which can more accurately guide the scaling of digital health platforms. For instance, in Rwanda, the facilitator observed interactions between external stakeholders and patients during the mCFIR session, where the stakeholder wanted to understand and hear feedback from the patient regarding their experience with the platform. In the Canadian Oak Tree Clinic, HCPs perceived security to be a concern to patients’ confidentiality and were surprised to hear from the patient during the mCFIR session that they had no security concerns related to partaking in the mHealth implementation. HCPs gave higher scores for Importance for the privacy and security constructs in comparison to patients. Concerns about confidentiality of patients information have been noted as a barrier to implementing eHealth interventions [23]. It is important to note however, that when a patient does not have a personal phone, privacy concerns arise, and this possibly explains why lack of access to phones was identified as an implementation barrier. Surprisingly though, the Importance score for the “accessibility” construct was relatively lower than the other constructs also associated to barriers of implementation. Access concerns have been noted elsewhere as barriers to implementation of mHealth interventions [24, 25].

Other constructs with relatively high mean Importance were training, user friendliness and language. Amongst health care providers, training is essential for implementing an intervention especially for those who may not have been present when the intervention was introduced. Partnership with health authorities and stakeholder engagement were repeatedly mentioned during the discussions. On the other hand, the outer setting Domain was rated the lowest for both Importance and Performance for all sites. We deduce that this could be a potential factor that hindered the scale-up of the WelTel platform. For an intervention to achieve scalability, we suggest investing efforts in the outer setting Domain.

We observed a parallel trend in the scoring of Performance and Importance of Domains. Domains that scored highest in Importance scored highest in Performance too. Accordingly, we speculate whether including only the Performance scale and leaving out the Importance scale would be enough for future mCFIR sessions as dichotomizing these scales may not be significant to participants.

The UBC mHealth Research Group is completing development of a publicly available data visualization tool to facilitate the analysis of the mCFIR inputs in order to provide immediate feedback at the end of the mCFIR session. This will allow participants to better identify the Domains and constructs that are performing well, and the ones that need focus, as well as provide further opportunity for discussion based on the visualized group responses. The mCFIR brings together diverse stakeholders to discuss all aspects of implementation from each of their diverse perspectives.

### Challenges

We identified several important challenges to our methodology. First, participant recruitment was a challenge within certain sites, and among different participant types. It was challenging to recruit patients, external stakeholders, and policy makers for the mCFIR session. We observed greater interest from health authorities and external organizations in East Africa to attend the mCFIR sessions with Rwanda having the highest external stakeholder participants. This is reflected by their success in scaling up their intervention to two additional sites [15]. There was a lack of patient representation in most of the Canadian sites. For instance, in Haida Gwaii, HCPs preferred to conduct the first mCFIR sessions without involving patients in order to test the mCFIR tool and ensure that questions would be relevant and appropriate for patients. Given the diversity of the participants, there is a possibility of power dynamics skewing the scoring and feedback shared by participants resulting in a social desirability bias [26]. Further limitations, including uneven distribution of participant types across sites, could have impacted the overall scoring. Convenience sampling may have potentially introduced an inherent selection bias that could impact the external validity of this study as participant recruitment was not random; participants were identified with the help of the clinics’ medical directors. With regards to the mCFIR tool, there are some limitations assumed to affect the overall scoring. This includes the possibility of participants misidentifying their roles in the survey, participants mis-ranking the Domains, and/or inaccurately distinguishing between the Importance and Performance scoring. There have been a number of missing entries where participants either left some answers blank or did not complete the survey due to time constraints. Language was also a barrier for some participants among the East African sites. Facilitators have offered translations to preferred languages upon request. There could also be a potential cross-cultural referencing, where certain constructs are perceived differently due to the diversity in cultures and needs of the different geographical and health contexts. Lastly, the approach required a significant amount of time for analysis. This significantly hindered the rapid process of feedback of results to the participants, which is an intended feature of the original CFIR tool, in order to adapt and inform implementation itself.

## Conclusion

This paper reports the first large-scale application of the mCFIR tool. The mCFIR tool was used to evaluate the implementation of an mHealth intervention across multiple global settings. This approach allowed for an improved understanding of the barriers and facilitators to the implementation and scale-up of the mHealth tool under investigation. We learned that HCPs are the most likely champions to the implementation of a digital health platform and that efforts are necessary to involve outer settings such as stakeholders and policy makers, which in turn facilitates scale up of mHealth interventions. Further site-specific analysis is currently underway. Results are being disseminated to the healthcare teams implementing the WelTel services in order to address the challenges mentioned. There have been suggestions around translating the mCFIR tool to accommodate for other languages. The tool will be reexamined to further revise the questions and potentially shorten the Domains to be more streamlined and operational. The mCFIR tool is currently in the planning phase for a second round at some of the sites presented in this paper for a time series analysis in order to assess progress in Performance across time, especially since some sites are implementing WelTel  as part of their current COVID-19 national pandemic response [27]. In parallel, the mHealth Research Group is completing the development of a publicly accessible data visualization tool to facilitate analysis so that facilitators can immediately share results with participants at the end of the mCFIR session; results are intended to be available to other implementation researchers.

## Supplementary Information

**Additional file 1**: mCFIR Tool, Survey

**Additional file 2**: mCFIR Constructs Table

**Additional file 3**: Health Issues and Implementation Goals by Site Table

## Data Availability

All data generated and/or analyzed during this study are available from the corresponding author on reasonable request.

## Abbreviations

mHealth: Mobile Health
SUS: Scale-Up and Spread
CFIR: Consolidated Framework for Implementation Research
mCFIR: Modified Consolidated Framework for Implementation Research
UBC: University of British Columbia
SMS: Short Message Service
HCP: Health Care Provider
BC: British Columbia
ANC: Antenatal Care Clinic
IMMC: Immunization Clinics
CCC: Comprehensive Care Clinics
WHC: Wamba Health Center
HIV: Human Immunodeficiency Virus
AIDS: Acquired Immunodeficiency Syndrome
NGO: Non-Governmental Organization
RBC: Rwanda Biomedical Centre
TB: Tuberculosis
LTBI: Latent Tuberculosis Infection
BCWH: British Columbia Women’s Hospital
SD: Standard Deviation
AMREF: African Medical and Research Foundation
ESRC: Ethics and Scientific Review Committee
Inc.: Incorporated
