# Supplementary material for: Evaluation of the implementation process of the mobile health platform ‘WelTel’ in six sites in East Africa and Canada using the modified consolidated framework for implementation research (mCFIR)
Source: BMC Med Inform Decis Mak. 2021 Oct 26;21:293. doi: 10.1186/s12911-021-01644-1 (PMC8546747; doi:10.1186/s12911-021-01644-1)
Supplement: Supplementary file 1 — Additional file 1: mCFIR Tool, Survey [file 12911_2021_1644_MOESM1_ESM.pdf]

Modified Consolidated Framework for Implementation Research (mCFIR)

Type of Stakeholder

What participant category do you fall under?

- ○ Policy Maker
- ○ External Stakholder
- ○ Client/Patient
- ○ Health Care Provider
- ○ WelTel Team Manager

An implementation science evaluation tool for health interventions

Introduction

This Tool was developed to assess the current or potential implementation of a health intervention. It is meant to be a collective effort between a team of individuals with diverse experience in using and implementing the intervention. The Tool can be used at any stage of development and/or implementation of an intervention - from pre-implementation planning to validation of intervention success to scale up efforts. The data can be used to compare across different interventions, the same intervention in different settings, or longitudinally to assess progress and to provide critical feedback on the path to scale over time. The mCFIR Tool utilizes the 5 domains outlined in the CFIR (Consolidated Framework for Implementation Research - Damschroder et al. 2009) plus an additional domain INFLUENTIAL to patients as users in health interventions.

${date://CurrentTime/PT}

Instructions

- Read each question and its description carefully.
- Discuss each question within the group and write the key points of your discussion in the **comment** section. Remember to keep the discussion focused and brief.
- Assign a post–discussion score from 1 to 10 on how well the intervention **performs** for each question.
- Assign a post–discussion score from 1 to 10 on how **important** this question is to the intervention’s implementation.
- The data will be compiled as a spreadsheet that can be transformed to other formats if necessary.

Participants

This activity is meant to be done in a single group session with a team of stakeholders. The team using this tool should include:

A facilitator, someone who is familiar with the use of this tool	At least one person who is involved with the outer setting (ie. government, external policies, other organizations, etc.)	At least one person who works in the implementer team. (ie: clinical director, program manager, etc.)	At least one end-user who is a health care provider	At least one end-user who is a patient

Glossary

- Domain 1: Intervention Characteristics Components and Background of the Intervention
- Domain 2: Outer Settings Outside the Implementer Team (government, external policies, other organizations, etc)
- Domain 3: Inner Settings Within the Implementer Team (hospital staff, company employees, etc.)
- Domain 4A: End-user characteristics Health Care Provider
- Domain 4B: End-user characteristics Patient
- Domain 5: Process Steps for Implementation

Domain 1: Intervention Characteristics - Components and Background

1. How well does the intervention perform in its intended setting?

ie: Does the intervention reach the most vulnerable populations and individuals in need of the service?

Please provide your comments here:
List what is working well and what needs improvement

Performance

	Failing		Needs Improvement			Neutral		Good			Excellent
	0	1	2	3	4	5	6	7	8	9	10
Please answer on a scale of 1 to 10.											

Importance: How important is it that the intervention addresses the current problem on a scale of 1 to 10?

	Not Important		Minimally Important		Somewhat Important		Important		Very Important	
	0	1	2	3	4	5	6	7	8	9	10

Please answer on a scale of 1 to 10

2. How adaptable is this intervention to meeting local needs ?

Please provide your comments here:
List what is working well and what needs improvement

Performance

Failing		Needs Improvement			Neutral		Good		Excellent	
0	1	2	3	4	5	6	7	8	9	10

Please answer on a scale of 1 to 10.

Importance: How important is it that the intervention can be adapted to meet local needs?

Not Important		Minimally Important		Somewhat Important			Important		Very Important	
0	1	2	3	4	5	6	7	8	9	10

Please answer on a scale of 1 to 10

3. How user-friendly is the service of this intervention?

ie: How steep is the learning curve to use it? How difficult is it to set up?

Please provide your comments here:
List what is working well and what needs improvement

Performance

	Failing		Needs Improvement		Neutral		Good		Excellent	
	0	1	2	3	4	5	6	7	8	9	10
Please answer on a scale of 1 to 10.											

Importance: How important is it that the intervention is user friendly on a scale of 1 to 10?

	Not Important		Minimally Important		Somewhat Important		Important		Very Important	
	0	1	2	3	4	5	6	7	8	9	10
Please answer on a scale of 1 to 10											

4. How advantageous is this intervention over alternative solutions that are already in place?

Please provide your comments here:
List what is working well and what needs improvement

Performance

	Failing		Needs Improvement		Neutral		Good		Excellent	
	0	1	2	3	4	5	6	7	8	9	10
Please answer on a scale of 1 to 10.											

Importance: How important is it that the intervention is advantageous over the alternative solutions already in place on a scale of 1 to 10?

	Not Important		Minimally Important		Somewhat Important		Important		Very Important	
	0	1	2	3	4	5	6	7	8	9	10
Please answer on a scale of 1 to 10											

5. How acceptable are the costs of the intervention to the implementer and users?

ie: Consider costs associated with implementation (investment, supply, opportunity costs, fees for use)

Please provide your comments here:
List what is working well and what needs improvement

Performance

	Failing		Needs Improvement			Neutral		Good			Excellent
	0	1	2	3	4	5	6	7	8	9	10

Please answer on a scale of 1 to 10.

Importance: How important is it that the costs of intervention are acceptable to implementer and users on a scale of 1 to 10?

	Not Important		Minimally Important			Somewhat Important		Important			Very Important
	0	1	2	3	4	5	6	7	8	9	10

Please answer on a scale of 1 to 10

Reflection: Intervention Characteristics

Suggestions for improvement:
Enter ways to improve upon *or overcome barriers identified in this domain*

Domain 2: Outer Setting - Outside Implementer Team

1. How well connected is the project implementer team with outer setting stakeholders?

ie: Example of outer setting stakeholders - MOH, WHO, regional health associations, etc.

Please provide your comments here:
List what is working well and what needs improvement

Performance

	Failing		Needs Improvement			Neutral		Good			Excellent
	0	1	2	3	4	5	6	7	8	9	10
Please answer on a scale of 1 to 10.											

Importance: How important is connectedness between the implementation team with outer setting stakeholders on a scale of 1 to 10?

	Not Important		Minimally Important			Somewhat Important		Important			Very Important
	0	1	2	3	4	5	6	7	8	9	10
Please answer on a scale of 1 to 10											

2. Do external stakeholders or competing organizations support the implementation of the intervention?

Please provide your comments here:
List what is working well and what needs improvement

Performance

	Failing		Needs Improvement			Neutral		Good			Excellent
	0	1	2	3	4	5	6	7	8	9	10
Please answer on a scale of 1 to 10.											

Importance: What level of importance does competition with external organizations using the intervention have on its scale up?

	Not Important		Minimally Important			Somewhat Important		Important			Very Important
	0	1	2	3	4	5	6	7	8	9	10
Please answer on a scale of 1 to 10											

These page timer metrics will not be displayed to the recipient.

First Click: *0 seconds*

Last Click: *0 seconds*

Page Submit: *0 seconds*

Click Count: *0 clicks*

3. How do external strategies contribute to spread this intervention?

ie: Governmental policies, recommendations, guidelines, collaborates, pay-for-performance, public benchmark reporting

Please provide your comments here:
List what is working well and what needs improvement

Performance

	Failing		Needs Improvement			Neutral			Good		Excellent
	0	1	2	3	4	5	6	7	8	9	10
Please answer on a scale of 1 to 10.											

Importance: How important do you think contribution from external strategies to spread this intervention is on a scale of 1 to 10?

	Not Important		Minimally Important			Somewhat Important			Important		Very Important
	0	1	2	3	4	5	6	7	8	9	10
Please answer on a scale of 1 to 10											

Reflection: Outer Setting

Suggestions for improvement:
Enter ways to improve upon or overcome barriers identified in this domain

Domain 3: Inner Setting - Within Implementer Team

1. How strong is the networking and communication within the target organization?

ie: Are staff well assimilated? Is there encouragement of peer collaboration? Is there open feedback and review among peers and across hierarchical levels? Is there high quality communication of mission and goals?

Please provide your comments here:
List what is working well and what needs improvement

Performance

	Failing		Needs Improvement			Neutral		Good			Excellent
	0	1	2	3	4	5	6	7	8	9	10
Please answer on a scale of 1 to 10.											

Importance: How important do you think strength in networking and communication within the target organization is on a scale of 1 to 10?

	Not Important		Minimally Important			Somewhat Important		Important			Very Important
	0	1	2	3	4	5	6	7	8	9	10
Please answer on a scale of 1 to 10											

These page timer metrics will not be displayed to the recipient.

First Click: *0 seconds*

Last Click: *0 seconds*

Page Submit: *0 seconds*

Click Count: *0 clicks*

2. Is use of the intervention welcomed, encouraged, and supported within the inner setting?

ie: To what extent does the implementer team feel like the pre-existing situation (without the intervention) needs change? Does the team agree with upper management on why the change is important? Does the organization and its leadership promote a learning climate to support the implementation process?

Please provide your comments here:
List what is working well and what needs improvement

Performance

	Failing		Needs Improvement			Neutral		Good			Excellent
	0	1	2	3	4	5	6	7	8	9	10
Please answer on a scale of 1 to 10.											

Importance: How important do you think welcoming, encouragement and support of the intervention within the inner setting is on a scale of 1-10?

	Not Important		Minimally Important			Somewhat Important		Important			Very Important
	0	1	2	3	4	5	6	7	8	9	10
Please answer on a scale of 1 to 10											

These page timer metrics will not be displayed to the recipient.

First Click: *0 seconds*

Last Click: *0 seconds*

Page Submit: *0 seconds*

Click Count: *0 clicks*

3. How well is the infrastructure and logistic support provided to the intervention by your organization?

ie: Troubleshooting, equipment fixes, etc.

ie: Consider technology infrastructure like internet connectivity, computer hardware, etc.

Please provide your comments here:
List what is working well and what needs improvement

Performance

	Failing		Needs Improvement			Neutral		Good			Excellent
	0	1	2	3	4	5	6	7	8	9	10
Please answer on a scale of 1 to 10.											

Importance: How important do you think logistic support provided to the intervention by your organization is on a scale of 1 to 10?

Not Important		Minimally Important		Somewhat Important			Important		Very Important	
0	1	2	3	4	5	6	7	8	9	10

Please answer on a scale of 1 to 10

These page timer metrics will not be displayed to the recipient.

First Click: *0 seconds*

Last Click: *0 seconds*

Page Submit: *0 seconds*

Click Count: *0 clicks*

Reflection: Inner Setting

Suggestions for improvement:
Enter ways to improve upon *or overcome barriers identified in this domain*

Domain 4A: End-User Characteristics - HCP

Definitions

Which health care providers are the end-users of the intervention?

1. The health care providers believe that the intervention improves health outcomes.

Please provide your comments here:
List what is working well and what needs improvement

Performance

	Failing		Needs Improvement			Neutral		Good			Excellent
	0	1	2	3	4	5	6	7	8	9	10
Please answer on a scale of 1 to 10.											

Importance: How important do you think belief in the intervention improving health outcomes is on a scale of 1 to 10?

	Not Important		Minimally Important			Somewhat Important		Important			Very Important
	0	1	2	3	4	5	6	7	8	9	10
Please answer on a scale of 1 to 10											

2. The health care providers feel well-trained and confident while using the intervention.

Please provide your comments here:
List what is working well and what needs improvement

Performance

	Failing		Needs Improvement			Neutral		Good			Excellent
	0	1	2	3	4	5	6	7	8	9	10
Please answer on a scale of 1 to 10.											

Importance: How important do you think training and confidence while using the intervention is on a scale of 1-10?

	Not Important		Minimally Important			Somewhat Important		Important			Very Important
	0	1	2	3	4	5	6	7	8	9	10
Please answer on a scale of 1 to 10											

3. The health care providers feel secure with the level of privacy offered by the intervention.

Please provide your comments here:
List what is working well and what needs improvement

Performance

	Failing		Needs Improvement			Neutral		Good			Excellent
	0	1	2	3	4	5	6	7	8	9	10
Please answer on a scale of 1 to 10.											

Importance: How important do you think comfortability with the intervention's privacy level is on a scale of 1 to 10?

	Not Important		Minimally Important			Somewhat Important		Important			Very Important
	0	1	2	3	4	5	6	7	8	9	10
Please answer on a scale of 1 to 10											

Reflection: End-User Characteristics - Health Care Providers

Suggestions for improvement:
Enter ways to improve upon *or overcome barriers identified in this domain*

Domain 4B: End-User Characteristics - Patients

1. Patients believe the intervention improves their health outcomes compared to the current practice.

Please provide your comments here:
List what is working well and what needs improvement

Performance

	Failing		Needs Improvement			Neutral		Good			Excellent
	0	1	2	3	4	5	6	7	8	9	10
Please answer on a scale of 1 to 10.											

Importance: How important do you think patient belief in the interventions ability to improve health outcomes is on a scale of 1 to 10?

	Not Important		Minimally Important			Somewhat Important		Important			Very Important
	0	1	2	3	4	5	6	7	8	9	10
Please answer on a scale of 1 to 10											

2. Patients feel confident in their own ability to use the intervention.

Please provide your comments here:
List what is working well and what needs improvement

Performance

	Failing		Needs Improvement			Neutral		Good			Excellent
	0	1	2	3	4	5	6	7	8	9	10
Please answer on a scale of 1 to 10.											

Importance: How important do you think patient confidence in their ability to use the intervention is on a scale of 1-10?

	Not Important		Minimally Important			Somewhat Important		Important			Very Important
	0	1	2	3	4	5	6	7	8	9	10
Please answer on a scale of 1 to 10											

3. Patients have adequate access to the service the intervention provides.

ie: Do they share the service with family or others in the community? Is there electricity access at home to charge an electronic device if the intervention requires changes?

Please provide your comments here:
List what is working well and what needs improvement

Performance

	Failing		Needs Improvement			Neutral		Good			Excellent
	0	1	2	3	4	5	6	7	8	9	10
Please answer on a scale of 1 to 10.											

Importance: How important do you think patient access to the intervention is on a scale of 1-10?

	Not Important		Minimally Important			Somewhat Important		Important			Very Important
	0	1	2	3	4	5	6	7	8	9	10
Please answer on a scale of 1 to 10											

4. Patients feel secure with the level of privacy offered by the intervention.

Please provide your comments here:
List what is working well and what needs improvement

Performance

	Failing		Needs Improvement			Neutral		Good			Excellent
	0	1	2	3	4	5	6	7	8	9	10
Please answer on a scale of 1 to 10.											

Importance: How important do you think patient security with the intervention's level of privacy is on a scale of 1 to 10?

Not Important		Minimally Important			Somewhat Important		Important			Very Important
0	1	2	3	4	5	6	7	8	9	10

	Not Important		Minimally Important		Somewhat Important		Important		Very Important	
	0	1	2	3	4	5	6	7	8	9	10
Please answer on a scale of 1 to 10											

5. Patients understand the language used in the intervention.

Please provide your comments here:
List what is working well and what needs improvement

Performance

	Failing		Needs Improvement		Neutral		Good		Excellent	
	0	1	2	3	4	5	6	7	8	9	10
Please answer on a scale of 1 to 10.											

Importance: How important do you think understanding the intervention's language is on a scale of 1 to 10?

	Not Important		Minimally Important		Somewhat Important		Important		Very Important	
	0	1	2	3	4	5	6	7	8	9	10
Please answer on a scale of 1 to 10											

Reflection: End-User Characteristics - Patients

Suggestions for improvement:
Enter ways to improve upon *or overcome barriers identified in this domain*

Domain 5: Implementation Process - Steps for Implementation

1. How adequate has attention been given to planning the implementation towards the set goal?

ie: Have steps been taken to promote change at organizational and individual levels to support implementation?

Please provide your comments here:
List what is working well and what needs improvement

Performance

	Failing		Needs Improvement			Neutral		Good			Excellent
	0	1	2	3	4	5	6	7	8	9	10
Please answer on a scale of 1 to 10.											

Importance: How important do you think strength in networking and communication within the target organization is on a scale of 1 to 10?

	Not Important		Minimally Important			Somewhat Important		Important			Very Important
	0	1	2	3	4	5	6	7	8	9	10
Please answer on a scale of 1 to 10											

2. How well are the stakeholders engaged during the implementation?

ie: Example of stakeholders - opinion leaders, formally appointment internal leaders, champions, external change agents, and user representatives.

Please provide your comments here:
List what is working well and what needs improvement

Performance

	Failing		Needs Improvement			Neutral		Good			Excellent
	0	1	2	3	4	5	6	7	8	9	10
Please answer on a scale of 1 to 10.											

Importance: How important do you think unity within the implementer team when making major changes is on a scale of 1-10?

	Not Important		Minimally Important		Somewhat Important		Important		Very Important		
	0	1	2	3	4	5	6	7	8	9	10
Please answer on a scale of 1 to 10											

3. How well has the intervention been carried out according to plan?

ie: Consider the following factors: Intensity of implementation, timelines of task completion, degree of engagement from key individuals.

Please provide your comments here:
List what is working well and what needs improvement

Performance

	Failing		Needs Improvement			Neutral		Good			Excellent
	0	1	2	3	4	5	6	7	8	9	10
Please answer on a scale of 1 to 10.											

Importance: How important do you think welcoming, encouragement and support of the intervention within the inner setting is on a scale of 1-10?

	Not Important		Minimally Important		Somewhat Important		Important		Very Important		
	0	1	2	3	4	5	6	7	8	9	10
Please answer on a scale of 1 to 10											

4. Is time dedicated for reflection or debriefing about the implementation before, during and after the implementation process?

ie: Is there a clear monitoring and evaluation plan in place?

Please provide your comments here:
List what is working well and what needs improvement

Performance

	Failing		Needs Improvement			Neutral		Good			Excellent
	0	1	2	3	4	5	6	7	8	9	10
Please answer on a scale of 1 to 10.											

Importance: How important do you think logistic support provided to the intervention by your organization is on a scale of 1 to 10?

	Not Important		Minimally Important			Somewhat Important		Important			Very Important
	0	1	2	3	4	5	6	7	8	9	10
Please answer on a scale of 1 to 10											

Reflection: Implementation Process

Suggestions for improvement:
Enter ways to improve upon *or overcome barriers identified in this domain*

Goal Attainment Scale

Goal Attainment Scale

Definitions

Restate your goals of this implementation.

How well are you achieving your implementation goals?

ie: Think about adoption, growth, and sustainability

Discussion

Performance

	Not yet meeting expectations		Approaching expectations			Meeting some expectations		Meeting all expecations		Exceeding expecations	
	0	1	2	3	4	5	6	7	8	9	10
Please answer on a scale of 1 to 10.											

Reflection: Goal Attainment

Suggestions for improvement:
Enter ways to improve upon *or overcome barriers to achieve the implementation goals.*

Impact Assessment

Impact Assessment

Definitions

Restate the key outcomes of this intervention.

How well are you achieving your key outcomes?

Discussion

Performance

	Not yet meeting expectations		Approaching expectations			Meeting some expectations		Meeting all expecations		Exceeding expecations	
	0	1	2	3	4	5	6	7	8	9	10
Please answer on a scale of 1 to 10.											

Reflection: Impact Assessment

Suggestions for improvement:
Enter ways to improve upon *or overcome barriers to match the key outcomes.*

THE UNIVERSITY OF BRITISH COLUMBIA

THE UBC SURVEY TOOL

Powered by Qualtrics
