# Supplementary material for: Evaluation of the implementation process of the mobile health platform ‘WelTel’ in six sites in East Africa and Canada using the modified consolidated framework for implementation research (mCFIR)
Source: BMC Med Inform Decis Mak. 2021 Oct 26;21:293. doi: 10.1186/s12911-021-01644-1 (PMC8546747; doi:10.1186/s12911-021-01644-1)
Supplement: Supplementary file 2 — Additional file 2: mCFIR Constructs Table [file 12911_2021_1644_MOESM2_ESM.docx]

Supplementary File 2: mCFIR Constructs

| **Domain Title** | **Construct Title** | **Construct Descriptive** |
| --- | --- | --- |
| **Domain 1 - Intervention Characteristics** | Performance Fidelity | **Construct 1**  How well does the intervention perform in its intended setting? |
| **Domain 1 - Intervention Characteristics** | Adaptability | **Construct 2**  How adaptable is this intervention to meeting local needs? |
| **Domain 1 - Intervention Characteristics** | User-friendliness | **Construct 3**  How user-friendly is the service of this intervention? |
| **Domain 1 - Intervention Characteristics** | Comparative advantage | **Construct 4** How advantageous is this intervention over alternative solutions that are already in place? |
| **Domain 1 - Intervention Characteristics** | Affordability | **Construct 5**  How acceptable are the costs of the intervention to the implementer and users? |
| **Domain 2 - Outer Setting** | Stakeholder Engagement | **Construct 6**  How well connected is the project implementer team with outer setting stakeholders? |
| **Domain 2 - Outer Setting** | External support | **Construct 7**  Do external stakeholders or competing organizations support the implementation of the intervention? |
| **Domain 2 - Outer Setting** | Scale-up support | **Construct 8**  How do external strategies contribute to spread this intervention? |
| **Domain 3 - Inner Setting** | Internal communication | **Construct 9**  How strong is the networking and communication within the target organization? |
| **Domain 3 - Inner Setting** | Acceptance | **Construct 10**  Is use of the intervention welcomed, encouraged, and supported within the inner setting? |
| **Domain 3 - Inner Setting** | Organizational Support | **Construct 11**  How well is the infrastructure and logistic support provided to the intervention by your organization? |
| **Domain 4A - End-User Characteristics, HCP** | Benefit Perception | **Construct 12**  The health care providers believe that the intervention improves health outcomes. |
| **Domain 4A - End-User Characteristics, HCP** | [HCP] Training | **Construct 13**  The health care providers feel well-trained and confident while using the intervention. |
| **Domain 4A - End-User Characteristics, HCP** | [HCP] Privacy | **Construct 14**  The health care providers feel secure with the level of privacy offered by the intervention. |
| **Domain 4B - End-User Characteristics, Patients** | Benefit Perception | **Construct 15**  Patients believe the intervention improves their health outcomes compared to the current practice. |
| **Domain 4B - End-User Characteristics, Patients** | [Pt.] Training | **Construct 16**  Patients feel confident in their own ability to use the intervention. |
| **Domain 4B - End-User Characteristics, Patients** | Accessibility | **Construct 17**  Patients have adequate access to the service the intervention provides. |
| **Domain 4B - End-User Characteristics, Patients** | [Pt.] Privacy | **Construct 18**  Patients feel secure with the level of privacy offered by the intervention. |
| **Domain 4B - End-User Characteristics, Patients** | Language | **Construct 19**  Patients understand the language used in the intervention. |
| **Domain 5 - Implementation Process** | Intervention planning | **Construct 20**  How adequate has attention been given to planning the implementation towards the set goal? |
| **Domain 5 - Implementation Process** | Stakeholder engagement | **Construct 21**  How well are the stakeholders engaged during the implementation? |
| **Domain 5 - Implementation Process** | Execution | **Construct 22**  How well has the intervention been carried out according to plan? |
| **Domain 5 - Implementation Process** | Evaluation | **Construct 23**  Is time dedicated for reflection or debriefing about the implementation before, during and after the implementation process? |
| **Goal Attainment Scale** | Goal attainment | **Construct 24**  How well are you achieving your implementation goals? |
| **Impact Assessment** | Outcome Assessment | **Construct 25**  How well are you achieving your key outcomes? |
