# Supplementary material for: Evaluation of the implementation process of the mobile health platform ‘WelTel’ in six sites in East Africa and Canada using the modified consolidated framework for implementation research (mCFIR)
Source: BMC Med Inform Decis Mak. 2021 Oct 26;21:293. doi: 10.1186/s12911-021-01644-1 (PMC8546747; doi:10.1186/s12911-021-01644-1)
Supplement: Supplementary file 3 — Additional file 3: Health Issues and Implementation Goals by Site Table [file 12911_2021_1644_MOESM3_ESM.docx]

Supplementary File 3: Health Issues and Implementation Goals by Site

| **Site** | **Health Issue Addressed Through Implementation of WelTel** | **Implementation Goals** |
| --- | --- | --- |
| Maralal, Kenya | 1) Continuity of care and access to healthcare provider support outside of regularly scheduled appointments/visits for pregnant women and children < 1.  2) Treatment support (i.e. medication, appointments, clinical instructions, immunization etc.) for pregnant women and children < 1 | 1) Reduce dropouts by 25% for antenatal care. 2) Include messages to increase Skilled Birth Attendance (SBA) at 36 weeks. 3) Include reminders for postnatal care. 4) Address transport and finance issues for clients who reply “Shida”. |
| Wamba, Kenya | 1) Continuity of care and access to healthcare provider support outside of regularly scheduled appointments/visits for pregnant women and children < 1.  2) Treatment support (i.e. medication, appointments, clinical instructions, immunization etc.) for pregnant women and children < 1. | Improving the following health indicators: 1) Improve antenatal Care. 2) Increase the rate of Skilled Birth Attendant at deliveries. 3) Improve adherence to child immunization. |
| Rwanda | 1) Side effects of treatment.  2) Follow up of treatment.  3) Inquire about clinic services.  4) Early detection of diseases.  5) Appointment reminders (for adolescents).  6) Psychosocial support.  7) Early management of treatment failure  8) Follow up on Prevention Mother to Child Transmission (PMTCT) | 1. Increase the percentage of viral suppression for adolescents from 86% to 97.8%.  2. Expand the WelTel platform to other health centers in Rwanda.  3. Sensitize patients to respond to the weekly check-in message. |
| Oak Tree, Vancouver | 1) Access to health care providers outside of regularly scheduled appointments. 2) Access to support surrounding Social Structural Factors / Social Determinants of Health 3) Adherence to treatment (i.e. medication, appointments, clinical instructions, etc.) for individuals living with HIV. | 1) Offer WelTel to all incoming patients; Enroll 100 new patients (receiving HIV treatment) in the OakTree Clinic in 1 year.  2) Obtain project funding to research the use of WelTel at OakTree, and its transferability to other patients/treatments in BC Women’s Hospital. |
| TB Clinic Vancouver | 1) Access to health care provider support outside of regularly scheduled appointments.  2) Treatment support (i.e. medication, appointments, clinical instructions, etc.) for TB patients. | 1) Offer WelTel to all incoming TB patients; enroll 100 new patients (receiving HIV treatment)  2) Obtain project funding to research the use of WelTel and its transferability to other patients/treatments. |
| Haida Gwaii, British Columbia | 1. Access to health care providers outside of regularly scheduled appointments 2. Initially targeting patients with one or more chronic disease, including: congestive heart failure (CHF), chronic obstructive pulmonary disease (COPD), cancer, diabetes mellitus, & hypertension. 3. Symptom assessment & management, data sharing, appointment reminders, appointment scheduling, prescription refills and adjustments, other (e.g. requisition forms, clinic hours). | 1) Work to integrate WelTel as an effective and sustainable primary care service in the clinic. Includes quality assurance of data in WelTel, training staff, and promote use among all practices to increase capacity and improve processes.  2) Change wording of “How Are You?” messaging to guide patients to use the service for principally medical needs.  3) Expand reach to patients with greatest need, but only once well-established and in consult with key implementers (i.e. Primary Care Assistants).  4) Continued training & support from WelTel for providers and implementation team.  5) Include other allied health on platform (i.e. physiotherapists, midwives). |
| Reach – proportion enrolled within your target population  Adoption – by Health Authority, by site, or by clinical practices (different levels)  Scale-Up – building infrastructure to support the full-scale implementation  Spread – replicating an intervention across populations or to different sites  Sustain/maintain – efforts to sustain the intervention in practice | | |
